# A Thermoelectric-Heat-Pump Employed Active Control Strategy for the Dynamic Cooling Ability Distribution of Liquid Cooling System for the Space Station’s Main Power-Cell-Arrays

**DOI:** 10.3390/e21060578

**Published:** 2019-06-10

**Authors:** Hui-Juan Xu, Ji-Xiang Wang, Yun-Ze Li, Yan-Jun Bi, Li-Jun Gao

**Affiliations:** 1 Advanced Research Center of Thermal and New Energy Technologies, Xingtai Polytechnic College, Xingtai, Hebei 054035, China; 2 School of Aeronautic Science and Engineering, Beihang University, Beijing 100191, China; 3 Institute of Engineering Thermophysics, North China University of Water Conservancy and Electrical Power, Zhengzhou, Henan 450045, China

**Keywords:** cold plate, temperature uniformity, thermoelectric heat pump, thermal management, control algorithm

## Abstract

A proper operating temperature range and an acceptable temperature uniformity are extremely essential for the efficient and safe operation of the Li-ion battery array, which is an important power source of space stations. The single-phase fluid loop is one of the effective approaches for the thermal management of the battery. Due to the limitation that once the structure of the cold plate (CP) is determined, it is difficult to adjust the cooling ability of different locations of the CP dynamically, this may lead to a large temperature difference of the battery array that is attached to the different locations of the CP. This paper presents a micro-channel CP integrated with a thermoelectric heat pump (THP) in order to achieve the dynamic adjustment of the cooling ability of different locations of the CP. The THP functions to balance the heat transfer within the CP, which transports the heat of the high-temperature region to the low-temperature region by regulating the THP current, where a better temperature uniformity of the CP can be achieved. A lumped-parameter model for the proposed system is established to examine the effects of the thermal load and electric current on the dynamic thermal characteristics. In addition, three different thermal control algorithms (basic PID, fuzzy-PID, and BP-PID) are explored to examine the CP’s temperature uniformity performance by adapting the electric current of the THP. The results demonstrate that the temperature difference of the focused CP can be declined by 1.8 K with the assistance of the THP. The proposed fuzzy-PID controller and BP-PID controller present much better performances than that provided by the basic PID controller in terms of overshoot, response time, and steady state error. Such an innovative arrangement will enhance the CP’s dynamic cooling ability distribution effectively, and thus improve the temperature uniformity and operating reliability of the Li-ion space battery array further.

## 1. Introduction

The Li-ion battery has been proven to be a promising candidate to substitute other energy storage batteries as the power source in space stations [1,2,3] owing to its merits of high power density, high single voltage, long cycling life, environmental friendliness, large operating temperature ranges and so on [4,5,6]. The operation performance of the Li-ion battery array greatly depends on its operating temperature and temperature uniformity. An improper operating temperature may contribute to the reduction in charging efficiency and service life of the batteries [7,8]. Further, an uneven temperature distribution of the single cells may potentially decrease the pack capacity and cause serious safety problems [9]. Accordingly, the battery thermal management system has become an essential approach to enhance the performance of the batteries effectively. 

The battery thermal management system has been investigated actively where different cooling technologies, namely air cooling, liquid cooling, heat pipe cooling, and phase change material cooling, were adopted. The air cooling approach [10,11] is classified into natural convection and forced air convection, and the latter is widely researched because of its high convective heat transfer coefficient. The heat pipe cooling [12,13] achieves heat transfer from the heat source to the cooling end to lower the battery array’s temperatur and is largely used in electrical devices for its high effective conductivity. As to the phase change material cooling method, the latent heat of the batteries is stored in the phase change material as the phase changes over a small temperature range, thereby the temperature rise inside the battery can be reduced [14,15]. When confronting more complicated configurations, especially in the case of a large-size high-rate discharging battery array [16], liquid cooling thermal management could manifest better performances than other methods. Furthermore, due to its advantages of strong heat dissipation ability, gravity immunity, structural simplicity, technological maturity, etc., the single-phase fluid loop is regarded as the most promising active approach for Li-ion battery thermal management in space stations. 

**Figure 1:**
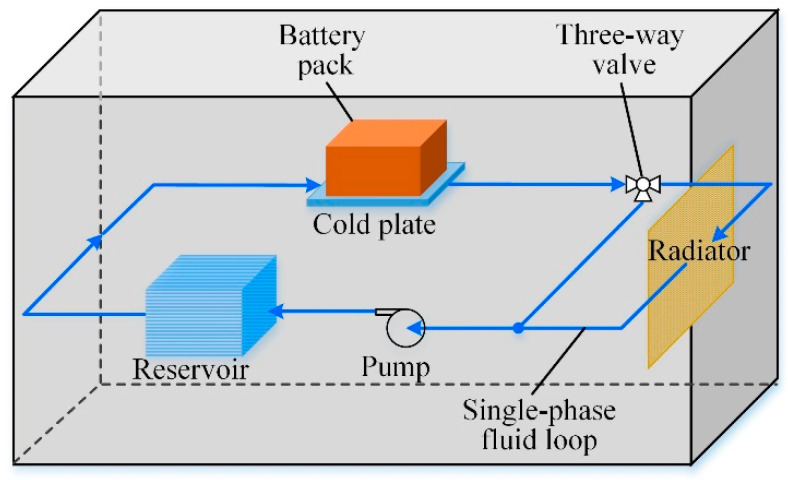
Traditional single-phase fluid loop with CP for Li-ion batteries.

The conventional strategy of the single-phase fluid loop for the space batteries [17] is shown in Figure 1. It comprises of the cold plate (CP), radiator, pump, reservoir and three-way valve. It is shown that the onboard vacuum packaged battery array is cascaded into a single-phase fluid cooling cycle system via a CP. The CP is applied for absorbing the heat generated inside the batteries and the coolant flowing through is for transporting heat to the radiator by which the heat is dissipated to the outer space. To balance the flow through the bypass line and the main fluidic line linking to the radiator, a three-way valve is used by adjusting the valve opening factor between 0% and 100%, through which the coolant temperature can be regulated. The reservoir collects the coolant flowing from the bypass line and the main line for the next cooling cycle, and acts as coolant supplement for the fluid loop when necessary. Undertaking a variety of works, including flow management, heat transfer and energy conversion, etc., the CP is considered to be the key component of the battery liquid cooling system on account of its compactness and ability to separate the battery and fluid [18,19,20].

Much attention has been devoted to the performance improvement of the CP. Yamada et al. [21] developed a honeycomb-cord CP that can obtain a remarkable mass decrease without lowering heat removal capability. Wang et al. [22] designed a silica-plates based cooling system for prismatic lithium-ion batteries during fast charging-discharging process and in specific operating conditions. Jarrett et al. [23] proposed a serpentine-channel CP for the battery liquid cooling system and assessed the effect from the geometry of the channels. The numerical results indicate that with the optimum design, both pressure drop and average temperature can be decreased, but at the expense of temperature uniformity. Wang et al. [24] presented an advanced single phase actively-pumped fluid loop using distributed thermal control strategy applied in spacecraft thermal control system, which included a self-driven CP and a paraffin-actuated thermal control valve. This self-driven control system not only simplified the structure of the conventional mechanically pumped fluid loop effectively, but also improved the operation economy significantly. 

**Figure 2:**
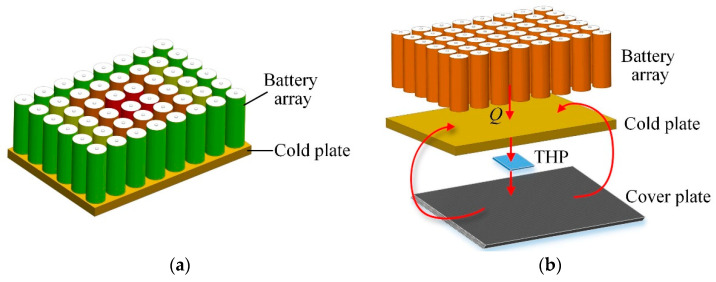
General design scheme. (**a**) temperature unevenness of space battery array with traditional single-phase fluid cooling; (**b**) temperature uniformity improvement of space battery array with proposed approach.

However, few studies focused on the dynamic adjustment of the cooling ability of CP’s different locations which is benefit for the improvement of the overall space battery’s temperature uniformity. As the structure of the CP is fixed, it is difficult to adjust the cooling capacity of the different locations of the CP dynamically which may cause the overheating area or undercooling area. Accordingly, this can directly lead to large temperature difference inside the cells, which is unfavorable for the reliability and capacity utilization of the battery array. As shown in Figure 2a, the temperature of the cells in the middle of the array is much higher than that of the cells on the edge of the CP, which can result in severe temperature unevenness of the power-cell-array and performance degradations or operating failures eventually. Therefore, it is urgent to investigate how to improve the temperature uniformity of the power-cell-array.

To achieve the dynamic cooling ability regulation of different locations of the CP, a thermoelectric heat pump (THP) and a high-heat-conductivity cover plate are employed in this study. Due to the small size, fast cooling speed, no environment pollution, and control simplicity, THPs have been extensively used in the temperature management system [25,26,27]. Traditionally, the cold side of the THP is attached to the cooling object and the hot side of THP the heat sink, which means that the cold-side temperature is usually the only variable can be controlled. The transferred heat in the hot-side heat is dissipated to the external environment space [28,29,30,31]. Nevertheless, different from conventional THP applications, the THP in Figure 2b functions as an effective heat transfer medium through which the heat can be transferred from the high temperature zone to the cover plate firstly. Then the accumulated heat in the cover plate is transferred to the low temperature zone of the CP through heat conduction. Through the function of the THP, the cooling ability of the different locations of the CP can be adjusted dynamically and the temperature uniformity of the power-cell-array can be enhanced notably.

In this paper, an active temperature uniformity control strategy of a novel combined CP-THP system (CCTS) is proposed to improve the temperature uniformity of the CP and finally, the Li-ion space array. A dynamic heat transfer model for the CCTS based on the lumped-parameter method is established. Simulation analyses are conducted to investigate the impacts of the heat load and THP’s electric current on the thermal characteristics of the CP. Three different controllers, namely the basic PID controller, fuzzy-PID controller, and BP-PID controller, are designed to manipulate the CP’s temperature difference by adjusting the electric current passing through the THP. With this THP-based active control strategy, CP’s maximum temperature difference can be decreased, and the temperature uniformity can be improved evidently, which implies that the temperature unevenness of the power-cell-array can be alleviated effectively as well. For further verification, relevant experiments have been conducted and the experimental work will be published in our subsequent paper.

## 2. System Description and Dynamic Modeling

### 2.1. General Idea of CCTS

**Figure 3:**
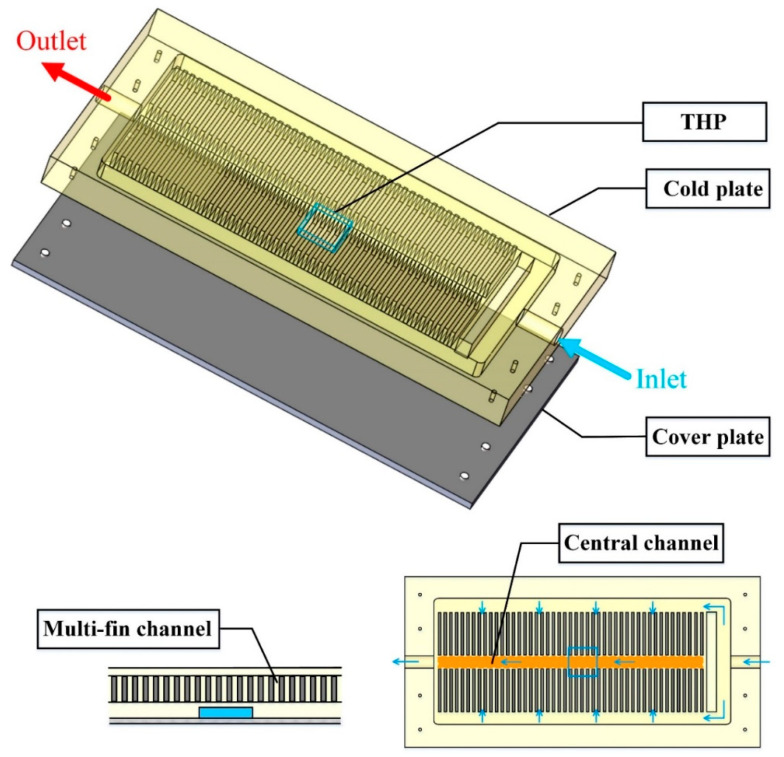
Construction details of the proposed CP module.

Usually, due to the non-uniformity of the flow pattern in the parallel channels, a clear non-uniform temperature distribution occurs in the CP accordingly which is unfavorable for the temperature uniformity of the space batteries. To alleviate this issue, an adaptive CP module based on the THP is put forward, as described in Figure 3.

The adaptive CP module comprises an aluminum CP, a THP embedded into the lower surface of CP, and a cover plate installed at the bottom of CP. Detailed geometric characteristics of the components of the CP modules are provided in Table 1. There are two kinds of channel in the CP: (1) multi-fin channels arranged symmetrically and (2) a central channel linking to the outlet. In operation, the working fluid flows from the inlet into the multi-fin channels of both sides. Then these two flows converge into the central channel to be charged out of the CP as a whole through the outlet. Specifically, the cover plate is made of carbon fiber because of its extremely thin thickness, light weight and excellent thermal conductivity, which is of great importance for the system operating in space. Additionally, to guarantee optimal contact among components, the cover plate with eight bolts is capable of sealing the CP perfectly during actual operation. 

**Figure 4:**
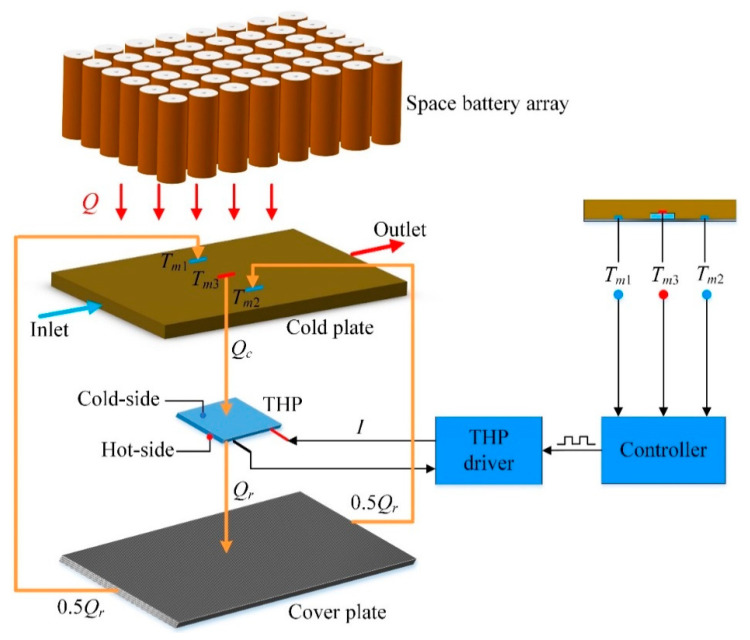
System configuration.

The configuration of the CCTS is exploded in Figure 4. As described in Figure 2a, the temperature of the batteries in the middle of the array is much higher than that of the batteries on the edge of the array for that the batteries in the middle manifest poor heat dissipation effect, which can also give rise to high temperature in the middle of the CP. Besides, according to the unique channel distribution of CP presented in Figure 3, theoretically there exists the high temperature area corresponding to the central channel in the middle and the low temperature area in the multi-fin channels. The above two reasons both result in temperature non-uniformity of the CP. It will harm the temperature uniformity of the batteries finally. To address this problem, a THP is embedded between the bottom of the CP and the top surface of the cover plate, as also shown in Figure 4, for transferring the heat from the high temperature zone to the low temperature zone of the CP via the carbon fiber plate. Specifically, the cold side of THP is attached to CP’s bottom surface while the hot side to the top surface of the cover plate. In theory, there should be temperature difference between the cold side and the hot side of THP due to the Peltier effect, which means that there may exist temperature difference between the CP and the cover plate. However, owing to the extremely high thermal conductivity and thin thickness of the cover plate, as well as the large contact area between the CP and the cover plate, this temperature difference can be reduced to be negligible. 

Considering mainly the effect of heat conduction, we define *Q* as the thermal load that the battery cells charges into the CP. For temperature acquisition in real time, three temperature sensors are placed on the bottom of CP, which are denoted in Figure 4 as *T_m_*_1_, *T_m_*_2_ and *T_m_*_3_ respectively. To be precise, *T_m_*_1_ and *T_m_*_2_ are temperatures of the multi-fin channels on both sides, *T_m_*_3_ is temperature of the outlet liquid-collecting channel in the middle. Theoretically, *T_m_*_1_ is identical with *T_m_*_2_ and both are less than *T_m_*_3_. Need of special note is that since the cold side of the THP is tightly attached to the middle area of CP’s lower surface, *T_m_*_3_ is considered to be equal to the cold side temperature of the THP.

The process of counterbalancing the heat is realized by manipulating the electric current that passes through THP. In operation, the electric current input to the THP is adjusted according to the temperature difference among *T_m_*_1_, *T_m_*_2_ and *T_m_*_3_. Therefore, an intelligent controller, as well as a THP driving unit, are designed for simulation.

### 2.2. Mathematical Modeling

In this section, working mechanism of the CCTS is provided from the point of view of the mathematics and thermodynamics including the lumped-parameter model and the controlling model, which may contribute to a better understanding of the function and dynamic characteristics of the system for the control of its thermal performance.

#### 2.2.1. Lumped-Parameter Model

**Figure 5:**
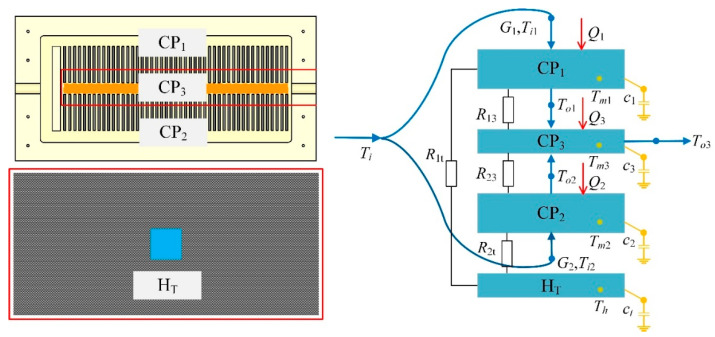
Lumped-parameter model.

As shown in Figure 5, a lumped-parameter-based dynamic heat transfer model for the CCTS is proposed to investigate the thermal performance of the CP in detail. In accordance with CP’s channel layout, the multi-fin channels on both sides can be treated as two lumped-parameter nodes which are CP_1_ and CP_2_ respectively, and the central channel is treated as the third lumped-parameter node CP_3_. Besides, the hot side of THP with the cover plate together is considered as the fourth lumped-parameter node H_T_ similarly, which will be fully described afterwards in this section.

For simplicity, some assumptions are made as follows: (1) the heat transferred from the battery cells to the CP is dominated by heat conduction denoted as thermal load *Q*; (2) the thermal resistance between the CP and carbon fiber plate is negligible considering the extremely thin thickness and excellent thermal conductivity of the cover plate, as well as large contacting area between the these two components; (3) the thermal resistance between the nodes of CP_1_ and CP_2_ is also inappreciable because of the small contacting area; (4) the thermal resistance between the node CP_3_ and the cold side of THP is considered to be zero for their optimal contact; (5) the initial temperature difference between the hot side and cold side of the THP is 1K.

According to Assumption (2) and Assumption (3), the temperature dynamics of the nodes of CP_1_, CP_2_, and CP_3_, which are based on the energy conservation principle, can be simplified and expressed by Equations (1)–(3).

(1) $$m_{1}c_{cp}\frac{dT_{m1}}{d\tau }=Q_{1}+\frac{1}{R_{13}}\left(T_{m3}-T_{m1}\right)+\frac{1}{R_{1t}}\left(T_{h}-T_{m1}\right)-c_{f}G_{1}\eta \left(T_{m1}-T_{i1}\right)$$

(2) $$m_{2}c_{cp}\frac{dT_{m1}}{d\tau }=Q_{2}+\frac{1}{R_{23}}\left(T_{m3}-T_{m2}\right)+\frac{1}{R_{2t}}\left(T_{h}-T_{m2}\right)-c_{f}G_{2}\eta \left(T_{m2}-T_{i2}\right)$$

(3) $$m_{3}c_{cp}\frac{dT_{m3}}{d\tau }=Q_{3}-\frac{1}{R_{13}}\left(T_{m3}-T_{m1}\right)-\frac{1}{R_{23}}\left(T_{m3}-T_{m2}\right)-c_{f}G_{3}\eta \left(T_{m3}-T_{i3}\right)-Q_{c}$$

where *m*_1_, *m*_2_ and *m*_3_ are the mass of the nodes of CP_1_, CP_2_ and CP_3_ respectively; *T_m_*_1_, *T_m_*_2_, *T_m_*_3_ and *T_h_* are the temperatures of the CP_1_, CP_2_, CP_3_ and the hot side of THP respectively; T*_i_*_1_, *T_i_*_2_ and *T*_i3_ are the inlet water temperatures for CP_1_, CP_2_ and CP_3_ respectively; *Q* is the thermal load of the battery cells; *c_cp_* and *c_f_* are the specific heat of the aluminum-made CP and water as working fluid; *R*_13_ and *R*_23_ are thermal resistances between CP_1_ and CP_3_, as well as between CP_2_ and CP_3_; *R*_1*t*_ and *R*_2*t*_ are thermal resistances between CP_1_ and the hot side of THP, as well as between CP_2_ and the hot side of THP; *G*_1_, *G*_2_ and *G*_3_ are the mass flow rates of CP_1_, CP_2_ and CP_3_ respectively; *η* is the heat exchange efficiency. It is noted that due to the symmetry of the two multi-fin channels, all the parameters of CP_1_ and CP_2_ are considered identical with each other theoretically (namely, *m*_1_ = *m*_2_, *T_m_*_1_ = *T_m_*_2_, *T_i_*_1_ = *T_i_*_2_, *G*_1_ = *G*_2_, *R*_13_ = *R*_23_, *R*_1*t*_ = *R*_2*t*_). Additionally, *G*_3_ is the total mass flow rate coming into the CP which is the sum of *G*_1_ and *G*_2_.

Equations (4) and (5) calculate the output water temperatures from the nodes CP_1_ and CP_2_ respectively. Equation (6) presents the temperature dynamic equation of the mixing working fluids from the nodes of CP_1_ and CP_2_. By solving the Equations (1)–(6), we can obtain the temperature of mixing water from nodes of CP_1_ and CP_2_ (*T_f_*_o_) which is actually the input water temperature of the node of CP_3_ (*T_i_*_3_) as expressed by Equation (7). Besides, the coolant outlet temperature from the CP is given by Equation (8).

(4) $$T_{o1}=T_{i1}+\eta \left(T_{m1}-T_{i1}\right)$$

(5) $$T_{o2}=T_{i2}+\eta \left(T_{m2}-T_{i2}\right)$$

(6) $$\begin{array}{ll} m_{f}c_{f}\frac{dT_{fo}}{d\tau } & =\left(G_{1}c_{f}T_{o1}+G_{2}c_{f}T_{o2}\right)-\left(G_{1}+G_{2}\right)c_{f}T_{fo}\\ & \;\;\;=G_{1}c_{f}\left(T_{o1}-T_{fo}\right)+G_{2}c_{f}\left(T_{o2}-T_{fo}\right) \end{array}$$

(7) $$T_{i3}=T_{fo}$$

(8) $$T_{o3}=T_{i3}+\eta \left(T_{m3}-T_{i3}\right)$$

The temperature dynamics of the node *H_T_* is represented by Equation (9). Therein, the heat flux *Q_h_* charged into the THP hot side can be estimated by Equation (10) and the cooling capacity *Q_c_* in the cold side of the THP which appears in the last term of the Equation (3) is given by Equation (11) where the *T_c_* is the cold-side temperature of the THP which is regarded as equal to that of the node of CP_3_ owing to Assumption (4). The power consumption of the THP is expressed by Equation (12). Given the cooling capacity *Q_c_* in Equation (11) and power supply *P* in Equation (12), the performance of THP can be evaluated by the coefficient of performance (COP) defined in Equation (13). A high COP means less power consumed by the THP, which is of great significance in simulating the CCTS for the operation of Li-ion batteries in the space stations.

(9) $$m_{t}c_{t}\frac{dT_{h}}{d\tau }=Q_{h}-\frac{1}{R_{1t}}\left(T_{h}-T_{m1}\right)-\frac{1}{R_{2t}}\left(T_{h}-T_{m2}\right)$$

(10) $$Q_{h}=\alpha_{t}\cdot T_{h}\cdot I+\frac{1}{2}I^{2}r_{t}-K_{t}\cdot \left(T_{h}-T_{c}\right)$$

(11) $$Q_{c}=\alpha_{t}\cdot T_{c}\cdot I-\frac{1}{2}I^{2}r_{t}-K_{t}\cdot \left(T_{h}-T_{c}\right)$$

where $\alpha_{t}$, *R*, and *K_t_* are the Seeback coefficient, electrical resistance, and the thermal conductivity of the THP, respectively.

(12) $$P=\alpha_{t}\cdot \left(T_{h}-T_{c}\right)\cdot I+I^{2}r_{t}$$

(13) $$\varepsilon =Q_{c}/P$$

Given a constant $\Delta T_{t}$ which is defined in Equation (14), the most suitable current corresponding to the peak value of COP can be attained by Equation (15) with the maximum COP gained by Equation (16) where the *M* can be acquired by Equation (17).

(14) $$\Delta T_{t}=T_{h}-T_{c}$$

(15) $$I_{\varepsilon_{\max}}=\frac{\alpha_{t}\Delta T_{t}}{\left(M-1\right)r_{t}}$$

(16) $$\varepsilon_{\max}=\frac{MT_{c}-T_{h}}{\Delta T_{t}\left(M+1\right)}$$

(17) $$M=\sqrt{1+0.5\left(T_{h}+T_{c}\right)}$$

#### 2.2.2. Control Model

The CCTS is a typical nonlinear system where there are difficulties in reducing the nonlinear constitutive equations to simple linear models while maintaining the accuracy of the respond of the system. To overcome such difficulties, three different control strategies which are basic PID control, fuzzy-PID control, and BP-PID control are proposed in this paper. The PID control serves as a base line for the comparative study with the other two intelligent control strategies. The temperature difference of CP (Δ*T_cp_*), obtained by Equation (18), is selected as the target variable based on which the control variable electric current passing through the THP is adjusted directly and the Δ*T_cp_* is further manipulated by changing heat flux *Q_h_* and cooling capacity *Q_c_* of the THP.

(18) $$\Delta T_{cp}=T_{m3}-T_{m1}=T_{m3}-T_{m2}$$

#### Basic PID Controller

**Figure 6:**
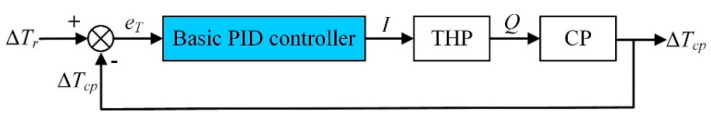
Block diagram of basic PID control system.

The block diagram of the CCTS with the basic PID controller is shown in Figure 6. As the input of the basic PID controller, the control error *e_T_* is the difference between the local temperature difference Δ*T_cp_* and the desired temperature difference Δ*T_r_* described in Equation (19). In this paper, the incremental PID control method is adopted for the basic PID controller, which can be expressed by Equation (20).

(19) $$e_{T}=\Delta T_{cp}-\Delta T_{r}$$

(20) $$\left\{ \begin{array}{l} \Delta u(t)=K_{p}\Delta e(t)+K_{i}e(t)+K_{d}\left[\Delta e(t)-\Delta e(t-1)\right]\\ \Delta e(t)=e(t)-e(t-1) \end{array}\right.$$

where *u*(*t*) is the incremental output of the controller at the sampling time *t*; *K_p_*, *K_i_*, *K_d_* are proportional coefficient, integral coefficient and differential coefficient, respectively; *e*(*t*) and *e*(*t*−1) are the deviation values at the sampling times *t* and *t*−1 respectively.

#### Fuzzy-PID Controller

**Figure 7:**
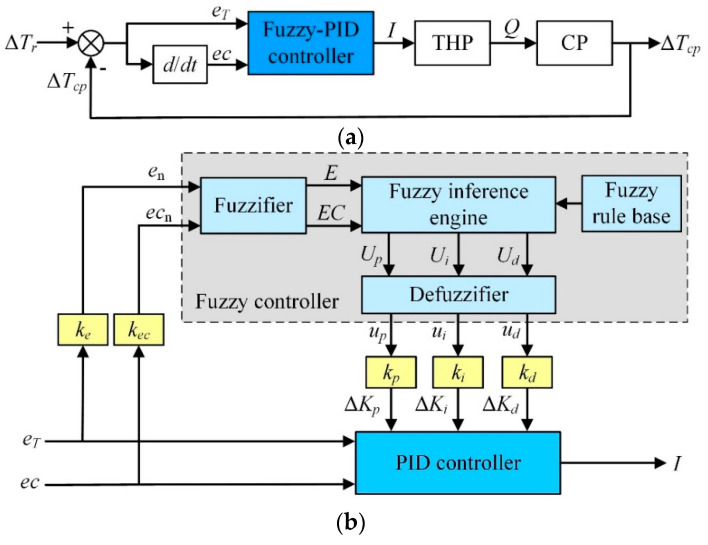
Block diagram illustrating CCTS with Fuzzy-PID controller; (**a**) Control block diagram and (**b**) Fuzzy-PID controller components.

Figure 7a shows the outline structure of the fuzzy-PID control system. On the basis of conventional PID controller, fuzzy-PID controller adopts the error *e_T_* and error change rate *ec* as inputs, and the parameters of *K_p_*, *K_i_*, *K_d_* as outputs. Figure 7b shows the detailed structure of the fuzzy-PID controller consisting of a fuzzifier, an inference engine, a defuzzifier, a fuzzy rule-base, and a PID controller. The inputs to the fuzzifier are the error *e_n_* and its changing rate *ec_n_* normalized by the factors *k_e_* and *k_ec_*. Similarly, the outputs of the defuzzifier *u_p_*, *u_i_*, and *u_d_* (scaled by the factors *k_p_*, *k_i_* and *k_d_*) are normalized increments of the controlling parameters *K_p_*, *K_i_* and *K_d_*. The relationships between the parameters (*K_p_*, *K_i_*, *K_d_*) and the inputs (*e_T_*, *ec*) can be expressed by Equation (21).

(21) $$\left\{ \begin{array}{l} K_{p}=K_{p0}+\{e_{T},ec\}k_{p}=K_{p0}+\Delta K_{p}\\ K_{i}=K_{i0}+\{e_{T},ec\}k_{i}=K_{i0}+\Delta K_{i}\\ K_{d}=K_{d0}+\{e_{T},ec\}k_{d}=K_{d0}+\Delta K_{d} \end{array}\right.$$

where *K_p_*_0_, *K_i_*_0_, and *K_d_*_0_ represent the initial values of *K_p_*, *K_i_* and *K_d_* respectively; *∆K_p_*, *∆K_i_*, and *∆K_d_* are the increments of *K_p_*, *K_i_* and *K_d_* respectively.

**Figure A1:**
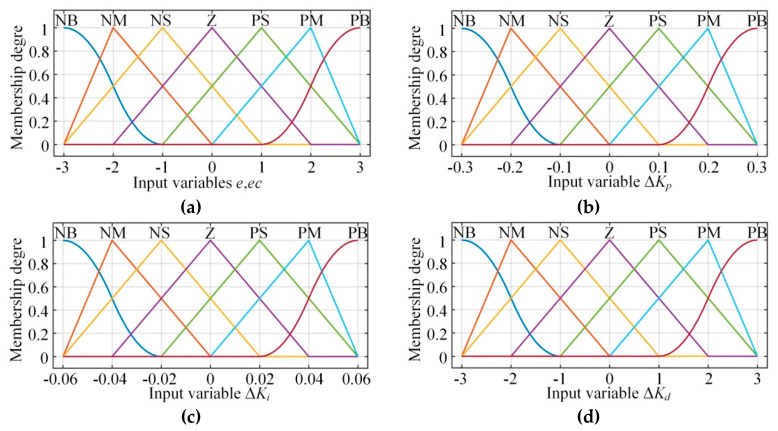
Membership functions; (**a**) *e_T_*,*ec*, (**b**) *∆K_p_*, (**c**) *∆K_i_*, and (**d**) *∆K_d_*.

The input and output variables of the fuzzy-PID controller are characterized by the fuzzy sets, linguistic values and associated analytical ranks which are listed in Table 2. Each fuzzy set (or its linguistic value) is defined by various membership functions shown in Figure A1.

The controller output is determined from the linguistic rules in the following form: if en is Ei and ecn is CEj, Then up is Ul(i,j), ui is Um(i,j), ud is Un(i,j). Ei, CEj, Ul(i,j), Um(i,j), and Un(i,j) are the fuzzy values of en, ecn, up, ui, and ud; and the subscript variables i, j, l(i,j), m(i,j) and n(i,j) denote the analytical ranks associated with these linguistic values listed in Table A1. For a two-input system (en and ecn, each with seven fuzzy values), a fully populated rule base will have 7 × 7 = 49 input rule combinations derived with the aid of simulations. 

#### BP-PID Controller

**Figure 8:**
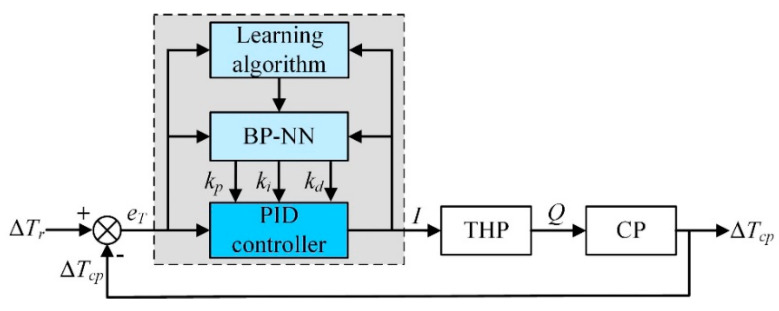
Control block diagram of BP-PID controller.

As Figure 8 illustrates, the back propagation PID (BP-PID) controller consists of two parts, the conventional PID controller and the back propagation neural network (BP-NN). The former controller carries on a direct closed-loop control of the THP with the online adjusted *K_p_*, *K_i_*, and *K_d_* obtained by the latter one.

**Figure 9:**
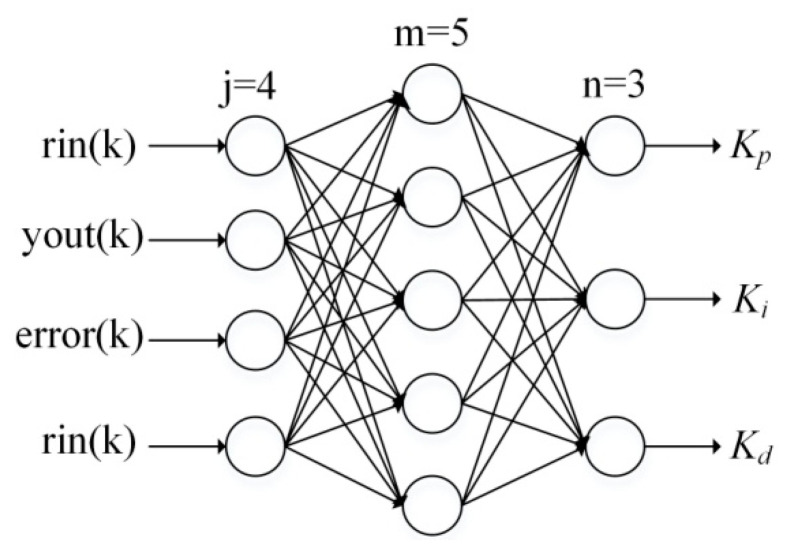
Structure of BP-NN.

A three-layer BP-NN with four input neuron nodes, five hidden nodes and three output nodes is set up, as shown in Figure 9. As inputs of the input layer, *rin*(*k*), *yout*(*k*), and *error*(*k*) adjust parameters of PID controller *K_p_*, *K_i_* and *K_d_* representing outputs of the output layer according to BP arithmetic.

Due to the input layer is equivalent model, the outputs are equal to the inputs, namely

(22) $$\left\{ \begin{array}{ll} O_{1}^{\left(1\right)}\left(k\right)=rin\left(k\right) & O_{2}^{\left(1\right)}\left(k\right)=yout\left(k\right)\\ O_{3}^{\left(1\right)}\left(k\right)=error\left(k\right) & O_{4}^{\left(1\right)}\left(k\right)=rin\left(k\right) \end{array}\right.$$

where *k* is the number of iterations, the superscript (1) refers to the input layer.

The inputs, activation function and outputs of the hidden layer can be expressed by Equations (23)–(25), where the superscript (2) represents the hidden layer, $\omega_{jm}^{\left(2\right)}$ is the connection weight from the input layer to the hidden layer defined in Equation (26), α is the learning rate, $\delta_{m}^{\left(2\right)}$ is the local gradient of the hidden layer that is given by Equation (27).

(23) $$net_{m}^{\left(2\right)}\left(k\right)=\sum_{j=1}^{4}\omega_{jm}^{\left(2\right)}O_{j}^{\left(1\right)}\left(k\right),\;m=1,2,3,4,5$$

(24) $$f(x)=\left(e^{x}-e^{-x}\right)/\left(e^{x}+e^{-x}\right)$$

(25) $$O_{m}^{\left(2\right)}\left(k\right)=f\left[net_{m}^{\left(2\right)}\left(k\right)\right]$$

(26) $$\Delta \omega_{jm}^{\left(2\right)}=\alpha \delta_{m}^{\left(2\right)}O_{j}^{\left(1\right)}\left(k\right)$$

(27) $$\delta_{m}^{\left(2\right)}=f^{\prime }\left(net_{m}^{\left(2\right)}\left(k\right)\right)\sum_{n=1}^{3}\delta_{n}^{\left(3\right)}\omega_{mn}$$

Similarly, the inputs, activation function and outputs of the output layer can be respectively written by Equations (28)–(30), where the superscript (3) represents the output layer, $\omega_{mn}^{\left(3\right)}$ is the connection weight from the hidden layer to the output layer which is deduced by Equation (31), $\delta_{n}^{\left(3\right)}$ is the local gradient of the output layer that can be expressed by Equation (32), and $d_{n}\left(k\right)$ is the desired output of the network. As previously mentioned, the output nodes of the output layer correspond to three adjustable parameters *k_p_*, *k_i_*, and *k_d_*, that is $O_{1}^{\left(3\right)}=k_{p}$, $O_{2}^{\left(3\right)}=k_{i}$, $O_{3}^{\left(3\right)}=k_{d}$. Since these parameters cannot be negative, the activation function of the output layer takes non-negative sigmoid function as Equation (29) describes.

(28) $$net_{n}^{\left(3\right)}\left(k\right)=\sum_{m=1}^{5}\omega_{mn}^{\left(3\right)}O_{m}^{\left(2\right)}\left(k\right),\;n=1,2,3$$

(29) $$g(x)=1/\left(1+e^{-x}\right)$$

(30) $$O_{n}^{\left(3\right)}\left(k\right)=g\left[net_{n}^{\left(3\right)}\left(k\right)\right]$$

(31) $$\Delta \omega_{mn}^{\left(3\right)}=\alpha \delta_{n}^{\left(3\right)}O_{m}^{\left(2\right)}\left(k\right)$$

(32) $$\delta_{n}^{\left(3\right)}=e_{n}\left(k\right)g^{\prime }\left(net_{n}^{\left(3\right)}\left(k\right)\right)=\left(d_{n}\left(k\right)-O_{n}^{\left(3\right)}\right)g^{\prime }\left(net_{n}^{\left(3\right)}\left(k\right)\right)$$

The learning rate is 0.7, and the inertia coefficient is 0.03 for simulation. The initial values of the connection weight coefficients distribute randomly on the interval from −1 to 1, as described in detail in Table A2.

#### 2.2.3. Solution Procedure and Simulation Condition Arrangement 

**Figure 10:**
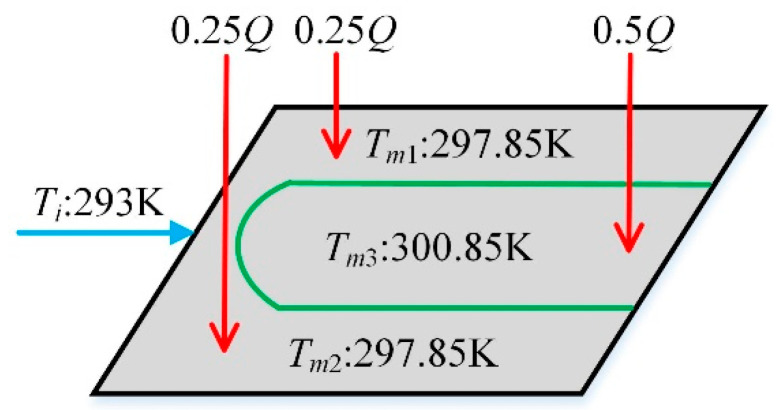
Partition setting of CP.

As shown in Figure 10, the CP is divided into three domains artificially corresponding to the three lumped-parameter nodes of the focused CP established in Section 2.2.1 (CP_1_, CP_2_, and CP_3_). To be more specific, the three parts account for 25%, 50%, and 25% of the total area of the CP. The initial temperatures of CP_1_, CP_2_, and CP_3_ are presented in Figure 10 as well, which are 297.85 K for CP_1_, 297.85 K for CP_2_, and 300.85 K for CP_3_ respectively. Notice that the initial temperature difference of CP (Δ*T_cp_*) is 3 K. The initial temperature of the THP are also preset as follows, in line with the partition setting of the CP. The cold-side temperature *T_c_* is set as 300.85 K which is identical with *T_m_*_3_ according to Assumption (4), and the hot-side temperature *T_h_* is set as 301.85 K according to Assumption (5).

Related parameter determinations of the CCTS and the initial state of the system are summarized in Table 3 where the thermal load of the batteries (*Q_i_*) and electric current of the THP (*I*) are critical input parameters as the main inputs for simulation. These two parameters are the primary variables that will be changed and controlled throughout the simulation process. As the base line for transient and control effect analyses, the initial *Q_i_* is 630W, and the initial *I* is 0 A. Note that in order to take the CP’s heat loss into account, we set the heat exchange efficiency of CP as 0.9.

**Figure 11:**
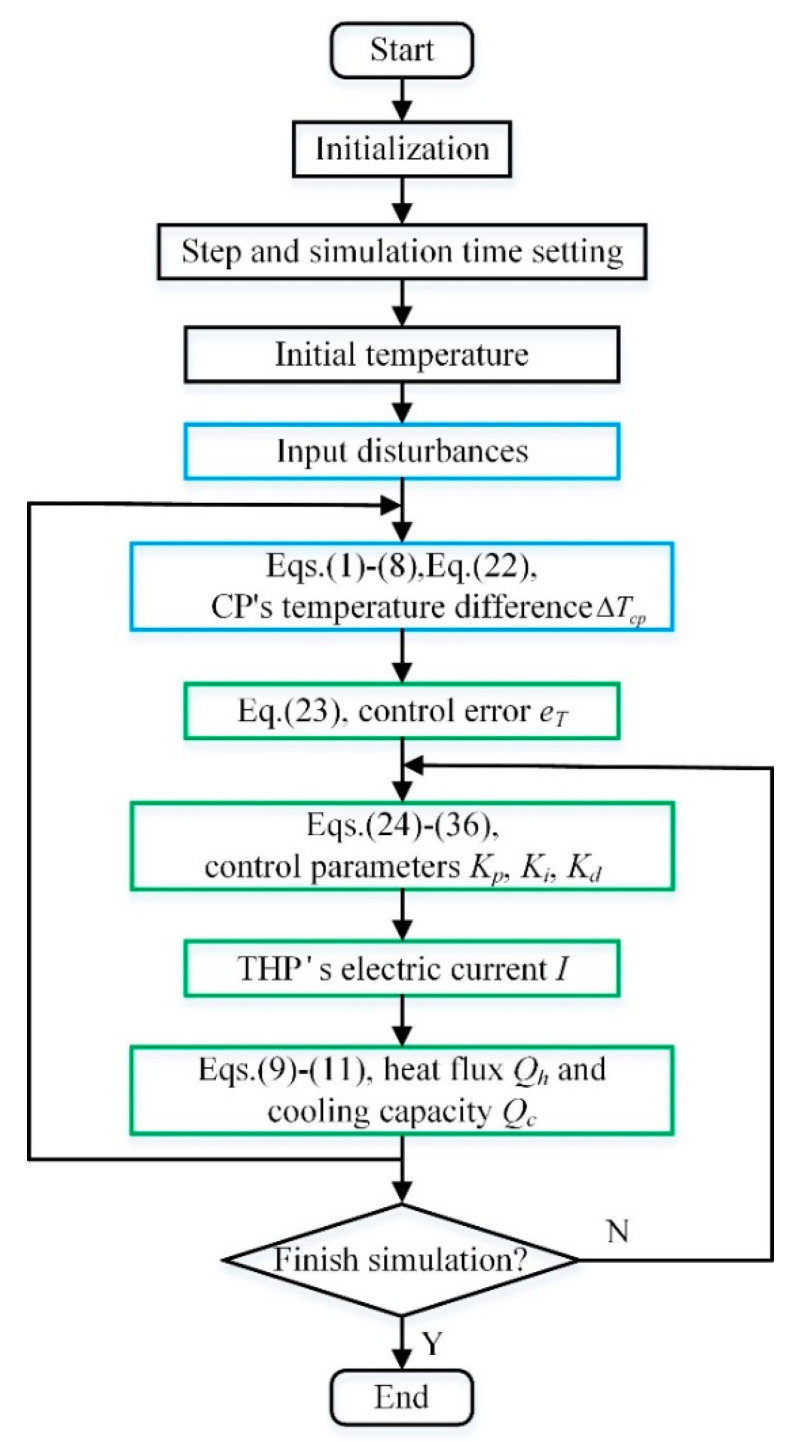
Simulation process flowchart.

Matlab R2017a was used as the simulation software. The flowchart of the simulation procedure is shown in Figure 11 which can be described in detail as follows: (1) The program is initialized at the beginning in terms of the physical and working-condition parameters listed in Table 3. (2) The simulation time and the calculation step are preset. (3) The initial temperatures of the CP and THP, *T_m_*_1_, *T_m_*_2_, *T_m_*_3_, *T_h_* and *T_c_* are given. (4) Various input disturbances tabulated in Table 4 are applied to the CCTS, beyond which the new value of Δ*T_cp_* can be derived according to Equations (1)–(8) and Equation (22). (5) During the closed-loop control, the control error *e_T_* can be obtained in terms of Equation (23) firstly; and then *e_T_* is transferred into different controllers to calculate the control parameters *K_p_*, *K_i_* and *K_d_* separately by Equations (24)–(36); afterwards the electric current *I* can be adjusted further; with the adjusted *I*, *Q_h_* and *Q_c_* brought to the CP will be computed using Equations (9)–(11), leading to a new Δ*T_cp_* finally. (6) After the above procedures, a judgment of whether the simulation time is over will be made. If the simulation time has not reached the set value and the control objective is un-convergence, the relative control algorithm should be modified to adapt the varying input disturbances. The simulation cycle will be updated by the new control parameters.

The focus of this study is to validate that the established CCTS can enhance CP’s temperature uniformity effectively. Two simulation conditions are arranged and listed in Table 4 accordingly. Specifically, a variety of step disturbances in the input *Q_i_* take place in the first simulation condition for demonstrating the influence of the heat load upon the temperature difference of CP. Step disturbances in the THP input current *I* occur in the second simulation condition to analyze the effect of the THP electric currents on CP’s temperature difference.

Additionally, to investigate the performance in rejecting disturbances of the designed controllers elaborated in Section 2.2.2, three cases (step disturbance, external disturbance and periodic disturbance) in *Q_i_* are organized and listed in Table 5. By evaluating the overshoot, settling time, and steady-state error together, we can evaluate the controlling performance of these three controllers when confronting different system disturbances. Determinations of the control parameters used in the simulation are summarized in Table A3. 

## 3. Open-Loop Dynamic Characteristics

The purpose of this section is to investigate the effects of the heat load *Q_i_* and electric current *I* of THP on the thermal characteristics of the CCTS. Due to the symmetry of multi-fin channels as stated in Section 2.1, *T_m_*_2_ changes identically with *T_m_*_1_ under certain condition and both have the same temperature. Therefore, *T_m_*_1_ is only discussed. The initial value of Δ*T_cp_* (*T_m_*_3_−*T_m_*_2_) is 3 K without the operation of the THP. On this basis, all the step-disturbances in *Q_i_* and *I* take place at 50 s.

### 3.1. Thermal Load Disturbance

**Figure 12:**
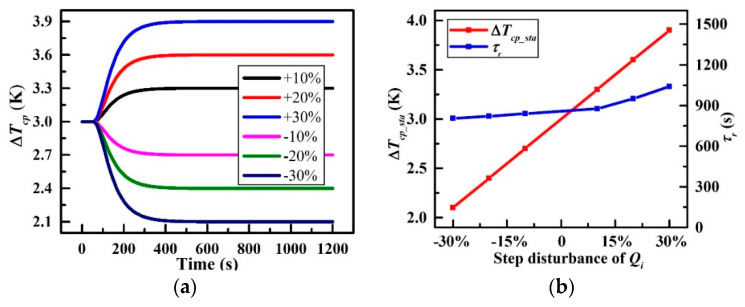
Characteristics under various step disturbances of *Q_i_*; (**a**) Δ*T_cp_* versus times and (**b**) Variations of Δ*T_cp_sta_* and *τ_r_* at different *Q_i_*.

Figure 12 demonstrates the characteristics of the CP under various step disturbances in the thermal load *Q_i_* corresponding to the first simulation condition in Table 4. Note that the heat load is 630 W at the first 50 s, followed by a variety of step-disturbances in an increment of 10% centred on the initial value. In Figure 12a, take the +30% step-disturbance for instance, Δ*T_cp_* rises rapidly from 3 K when the step-disturbance occurs, then it settles to be 3.9 K ultimately which is represented by the blue. For other positive step-disturbance cases, Δ*T_cp_* presents the same trend as well. In contrast, the trend is the opposite for the negative step-disturbance cases. CP’s stable temperature difference (Δ*T_cp_*__*sta*_) and the response time *τ_r_* at different *Q_i_* are plotted in Figure 12b. It is obvious that Δ*T_cp_*__*sta*_ raises linearly at a rate of 4.76 × 10^−3^ K/W with a slight increase in *τ_r_* from 800 s to 1100 s with the increase in *Q_i_* from −30% step-disturbance to +30% step-disturbance. The above observations suggest that more heat load applied to the CP leads to a more severe temperature non-uniformity and a longer settling time. In addition, the temperature difference of the CP is proportional to the heat load.

### 3.2. Electric Current Disturbance

**Figure 13:**
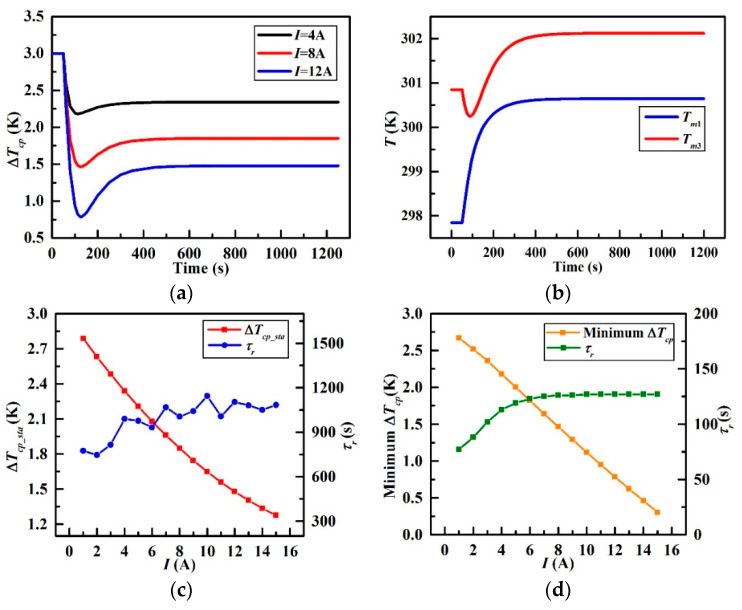
Characteristics of different step changes in I; (**a**) Δ*T_cp_* versus times, (**b**) Temperature variations at the 12 A step-disturbance, (**c**) Δ*T_cp_sta_* and *τ_r_* versus *I*, and (**d**) Minimum Δ*T_cp_* and *τ_r_* versus *I*.

Figure 13 presents the effect of THP electric current on the temperature changes of the CP. Figure 13a shows three typical step-disturbances of the current (4 A, 8 A and 12 A) upon the Δ*T_cp_*. It is obvious that Δ*T_cp_* in these three cases goes down rapidly to the minimum at about 120 s followed by a slow rise, and then attains a new level of equilibrium finally. The new Δ*T_cp_* is lower than the initial one of 3K. 

Figure 13b illustrates the transient temperature curve of *T_m_*_1_ and *T_m_*_3_ under the step-disturbance of 12 A. It can be observed that both *T_m_*_1_ and *T_m_*_3_ increase in that the power consumption of the THP will be transferred into waste heat which will be applied to the CP finally. To be specific, *T_m_*_1_ rises sharply and settles to a new steady-state value of 300.65 K under the 12 A step-disturbance at 50 s. Nevertheless, *T_m_*_3_ declines in a small range at the beginning and escalates to a new stable value of 302.12 K because of *Q_c_* removed from the node of CP_3_. Therefore, Δ*T_cp_* reaches a minimum at first and gets to a new stable level finally, which explains that why there exist valley in Figure 13a.

The range of the current step-disturbance is from 1 A to 15 A with the increment of 1 A, Δ*T_cp_sta_* varies from 2.79 K to 1.28 K accordingly in Figure 13c where the relationship between Δ*T_cp_sta_* and *I* suggests an approximately negative proportional relationship while the steady-state time *τ_r_* ranges slightly from 700 s to 1200 s and exhibits an increasing trend as a whole. Meanwhile, minimum Δ*T_cp_* in Figure 13d declines from 2.67 K to 0.8 K linearly with the climbing THP current from 1 A to 15 A. Furthermore, the time for minimum Δ*T_cp_* increases quickly at first from 75 s and the increasing rate becomes slow when the time comes to 125 s.

The above observations demonstrate that the THP has a significant effect on the temperature uniformity of the CP absolutely, which is the biggest discovery and innovation in this study. The temperature difference can be reduced by 1.8 K under the maximum current step-disturbance of 15 A of the THP in this paper. However, the higher the current is, the more power the system will consume as expressed in Equation (12). This means more waste heat will be generated which leads to an increase in the overall temperature of the CP. It should be acknowledged that such side-effect is not favorable for the system operation and should be minimized. Therefore, additional discussions in Section 3.3 were carried out to investigate the optimum operating condition of the THP in terms of small temperature difference of the CP and optimum COP of the THP.

### 3.3. Optimum Operating Conditions of THP

This section aims to determine the optimal range of the control variable *I* considering the optimal COP with small power consumption which facilitates to depress the excursion of the overall temperature of the CP as shown in Figure 13b, as well as the Δ*T_cp_* which should be small that is crucial for the CCTS.

Before the simulation results being discussed, correlative calculations theoretically should be carried out for further comparison. According to Equations (14) and (15), and the listed parameters of the THP listed in Table 3, the calculated maximal COP $\varepsilon_{\max}$ is 0.978 and the calculated optimal current for this maximal COP $I_{\varepsilon_{\max}}$ is 0.996 A on the condition that the ∆*T_t_* remains unchanged of 1K in simulation according to Assumption (5). 

**Figure 14:**
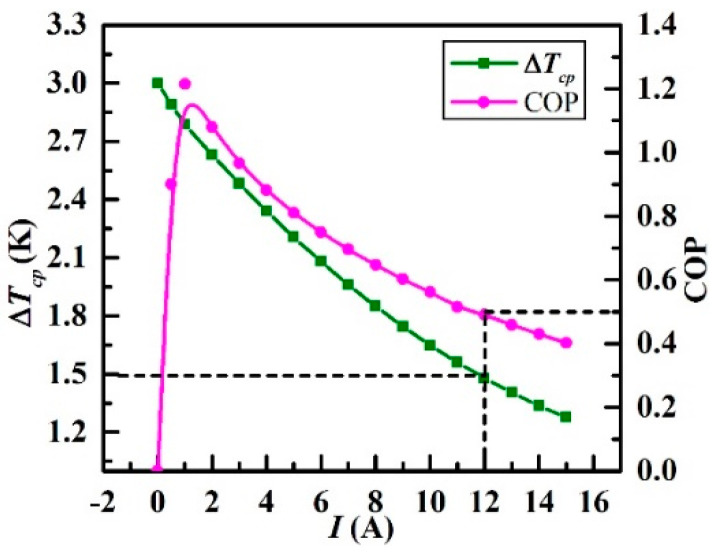
Variations of Δ*T_cp_* and COP under different currents.

Simulation results plotted in the form of Δ*T_cp_* and COP versus *I* in the range of 0–15 A are presented in Figure 14. It can be seen that Δ*T_cp_* declines gradually with the increasing electric current. The minimum temperature difference is 1.2 K with the maximum current of 15 A. Addtionally, the COP increases rapidly for a small range of the current (0–1 A) and the COP reaches its peak (1.2) when the current is 1 A. After that, the COP decreases gradually to final about 0.4 which is the lowest when the current is 15 A. This simulation result presents a good agreement with the theoretical optimal values ($I_{\varepsilon_{\max}}=0.996\;A$ and $\varepsilon_{\max}=0.978$).

The observations suggest that higher current (no more than 15 A) results in smaller temperature difference while lower current can obtain a higher COP when the current is within 1–15 A. In this paper, we propose scrupulously that 0.5 is the minimum acceptable COP in the practical operation. Therefore, the THP current is recommended to be controlled below 12 A, and the minimum temperature difference is 1.5 K. On this basis, three strategies are proposed in Section 4 to control the electric current well above a certain COP level (0.5 in this paper) while improving the temperature uniformity of the CP at the same time.

## 4. Closed-Loop Control Effect Analyses

In this section, the CCTS with three controllers, which are Basic PID, fuzzy-PID, and BP-PID controllers, responses to a variety of disturbances in the thermal load for closed-loop simulation. The results under these three control strategies, which examine the closed-loop control effects, are compared from the parameters such as overshoot, settling time, and steady-state error. 

Notice that the desired temperature difference ∆*T_r_* in the closed loop is set to be 1.5 K in this study in that when operated under the current of 12 A as stated in Section 3.3, Δ*T_cp_* was reduced by 1.5 K (from 3 K to 1.5 K). For simplicity, the dynamic temperature difference responses to manipulations from basic PID controller, fuzzy-PID controller, and BP-PID controller are defined as ∆*T_cp_*__PID_, ∆*T_cp_*__F_ and ∆*T_cp_*__BP_ respectively.

### 4.1. Step Disturbance Response

**Figure 15:**
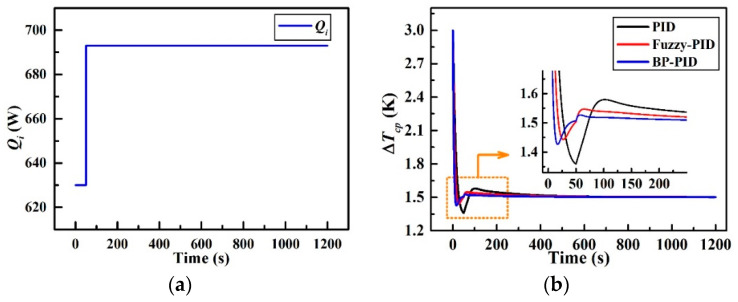
Simulated responses to a step-disturbance in *Q_i_*; (**a**) +10% step-disturbance in *Q_i_* and (**b**) transient curve of ∆*T_cp_*.

The dynamic temperature difference responses to a 10% step increase in the input heat load *Q_i_* to the CCTS are plotted in Figure 15. As mentioned in Section 3.1, ∆*T_cp_* will climb and settle to a new steady-state value of 3.3 K (0.3 K higher than initial 3 K) without closed-loop control under a +10% step-disturbance in the heat load *Q_i_* at 50 s. As shown in Figure 15b, all the three controllers achieve the objective of 1.5 K. However, there are several specific differences in the control effects among these three control strategies. The closed-loop overshoots *γ*, settling time *τ*, and steady-state errors *δ* of the simulated transients are summarized in Table 6. The overshoot, settling time, and steady-state error of the basic PID controller are calculated to be 9.4% (0.14 K), 68 s and 0.426% (0.006 K) respectively as a reference for the comparison. Obviously, the fuzzy-PID controller and BP-PID controller offer a much better temperature dynamic performance than the reference. The overshoot of ∆*T_cp_*__F_ is reduced to 39.1% compared with that of ∆*T_cp_*__PID_, and the settling time in ∆*T_cp_*__F_ is shortened by 52 s compared with that of ∆*T_cp_*__PID_. The overshoot of ∆*T_cp_*__BP_ is reduced to 52.4% compared with that of ∆*T_cp_*__PID_, and the settling time is accelerated by 58 s compared with that of ∆*T_cp_*__PID_. Besides, the steady-state errors of both ∆*T_cp_*__F_ and ∆*T_cp_*__BP_ are all sufficiently small which can be neglected according to the values in Table 6. Generally, BP-PID controller is better than fuzzy-PID controller from the perspective of the response rate and system stability. In contrast, the latter performs better from the perspective of the maximum overshoot.

### 4.2. External Disturbance Response

**Figure 16:**
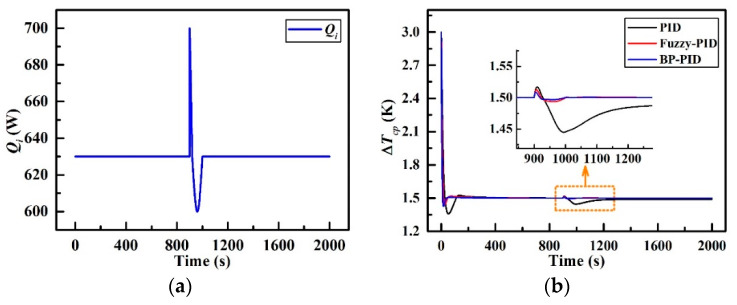
Effect of external disturbance in *Q_i_*; (**a**) *Q_i_* with external disturbance and (**b**) Responses of ∆*T_cp_*.

The transient responses of the temperature difference to an unfavorable external disturbance in *Q_i_* are depicted in Figure 16. It can be viewed from Figure 16a that there is a sharp wave pulse disturbance and a half-sinusoid pulse disturbance in the heat load brought from battery cells within 900–1000 s, which may be attributed to the abrupt change in the discharge rate of the Li-ion batteries [32,33,34]. The peak of the sharp wave pulse is 700 W at 900 s and the valley of the half-sinusoid pulse is 600 W at 960 s. As shown in Figure 16b, once the disturbance occurs at 900 s, ∆*T_cp_*__PID_ manifests a fluctuation (maximum overshoot of 0.017 K at 909 s) to the sharp pulse at first and then, a major oscillation (maximum overshoot of 0.055 K at 995 s) to the half-sinusoid pulse, followed by a slow returning at 1000–1300 s. ∆*T_cp_*__F_ and ∆*T_cp_*__BP_, nevertheless, display just a tiny fluctuation (maximal overshoot of 0.012 K at 907 s and 0.008 K at 905 s respectively) to the sharp pulse with a shorter returning time (20 s and 18 s respectively). In addition, there are no significant responses to the half-sinusoid pulse for these latter two control strategies. The corresponding parameters are listed in Table 7. ∆*T_cp_*__F_ manifests the least maximum overshoot which is 3.81% (0.06 K) while ∆*T_cp_*__BP_ provides the fastest settling time of 10 s compared with that of the other two. With regard to the steady-state performance, the steady-state errors of ∆*T_cp_*__F_ and ∆*T_cp_*__BP_ are both infinitesimally small, and so can be ignored. Therefore, it can be concluded that the fuzzy-PID controller and BP-PID controller present better temperature dynamic ability in responding to unexpected external disturbances as compared to the basic PID controller. 

### 4.3. Periodic Disturbance Response

**Figure 17:**
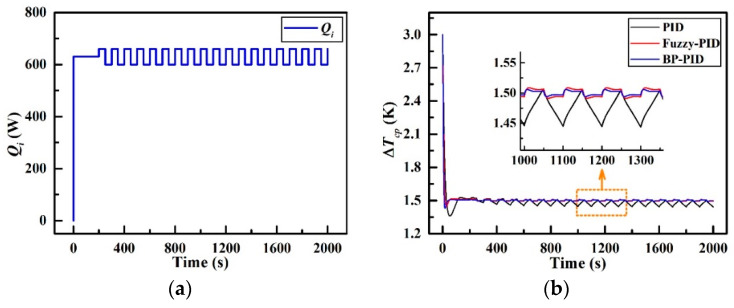
Temperature responses in the periodical heat load disturbance; (**a**) *Q_i_* with periodic disturbance and (**b**) ∆*T_cp_* variations.

Figure 17 presents the dynamic temperature difference responses to a periodic disturbance in *Q_i_*. As revealed in Figure 17a, the periodic disturbance in *Q_i_* is simulated in the form of a central constant value of 630 W at the first 200 s and a square wave pulse with high level of 660 W and low level of 600 W respectively after 200 s. The results can be found from Figure 17b that ∆*T_cp_*__PID_ fluctuates within 4% of its steady-state value (the absolute overshoot is 0.06 K) with the settling time of 84 s, while ∆*T_cp_*__F_ fluctuates within 0.53% of its steady-state value (the absolute overshoot is 0.008 K) with the settling time of 16 s. Further, ∆*T_cp_*__BP_ reaches an equilibrium after 10 s with a merely 0.4% fluctuation of its steady-state value (the absolute overshoot is 0.006 K). Additionally, the maximum overshoots of these three controllers are 9.31% (at 53 s), 3.6% (at 27 s), and 4.7% (at 17 s) respectively, and the settling times are 84 s, 16 s and 10 s respectively. All the parameters above can be referred to Table 8 for comparison. The observations suggest that when experiencing a periodical disturbance in the heat load, both fuzzy-PID controller and BP-PID controller still manifest fast response ability and strong self-adaptability as previously expected.

## 5. Conclusions

For the purpose of actively adjusting the cooling ability of different locations of the cold plate (CP), which is a key component of the single-phase fluid loop, a combined CP-THP system (CCTS) comprising a micro-channel CP integrated with a thermoelectric heat pump (THP) for thermal management of the Li-ion space battery array is proposed to improve the temperature uniformity of the space batteries. The adoption of the THP, which is intended to balance the internal heat transfer within the CP by regulating the THP electric current, is the largest innovation in this paper. The dynamic model for the evaluation of the CP’s thermal characteristics is theoretically established and three control strategies aiming to achieve effective and robust control of the temperature difference within the CP confronting various disturbances are developed. Numerical analyses for both open-loop and closed-loop simulations under different working conditions have been illustrated. Primary conclusions are summarized as follows.

(1) The maximum temperature difference of the CP was largely influenced by the heat load. Increasing the heat load from the batteries aggravated CP’s temperature unevenness and lengthened the settling time.
(2) The THP may dynamically adjust the cooling ability of the different locations of the CP and significantly improve the temperature uniformity of the CP under active control strategies. Compared with the traditional module without the THP, the temperature difference of the CP was decreased by 1.8 K with the maximum electric current (15 A) of the THP. The higher electric current was, the better temperature uniformity of the CP can be obtained. Nevertheless, a high electric current may result in unnecessary waste heat which will increase the overall temperature of the CP. Considering the tradeoffs of the temperature difference and power consumption, the electric current should be controlled below 12 A.
(3) Confronting various conditions of step disturbance, external disturbance and periodic disturbance, both fuzzy-PID and BP-PID controllers achieve an excellent control ability with sufficient fast response and strong stability, which are attractive alternatives to the basic PID controller. Specifically, the fuzzy-PID controller specializes in decreasing overshoot while the BP-PID controller facilitates a reduction of the response time and steady state error.

In conclusion, this THP-based temperature uniformity controlling system can achieve the cooling ability readjustment of different locations of the focused CP, which is extremely benefit for the temperature uniformity improvement of the space battery array. It is expected that this approach would have promising application prospects for other thermal control systems where extremely strict temperature uniformity demands should be satisfied.

## Figures and Tables

**Table 1:** Geometric parameters of CP module.

Parameters (mm)		Value
**Cold plate**	Outline Size (length × width × height)	600 × 400 × 16
	Wall Thickness	6
	Channel Width	5
	Channel Interval	5
	Channel Height	10
**Cover plate**	Outline size (length × width × height)	600 × 400 × 2
**THP**	Outline size (length × width × height)	40 × 40 × 4

**Table 2:** Fuzzy sets and their linguistic values.

Fuzzy Sets	Ranks	Linguistic Values
NB	−3	negative high
NM	−2	negative medium
NS	−1	negative low
Z	0	zero
PS	1	positive low
PM	2	positive medium
PB	3	positive high

**Table 3:** Parameter determination of the CCTS.

Parameter (Unit)	Symbol	Value
*Cold-plate*		
Thermal load (W)	*Q_i_*	630
Mass (kg)	*m_cp_*	6.8
Specific heat (J∙kg^−1^∙K^−1^)	*c_cp_*	924
Heat exchange efficiency	*η*	0.9
Heat resistance (K/W)	*R*_13_, *R*_23_	0.171
	*R*_1t_, *R*_2t_	0.067
*Working fluid*		
Mass (kg)	*m_f_*	0.828
Specific heat (J∙kg^−1^∙K^−1^)	*c_f_*	4200
Mass flow rate(kg/s)	*G*	0.02
Inlet temperature (K)	*T_i_*	293
*THP*		
Electric current (A)	*I*	0
Seeback coefficient (W/K/A)	*α_t_*	0.052
Electrical resistance (Ω)	*r_t_*	0.9
Thermal conductance (W/K)	*K_t_*	1.182
Heat capacity (J/K).	*m_t_c_t_*	619.8

**Table 4:** Case arrangement for open-loop dynamic analysis.

Simulation Conditions	Inputs	Final Value	Description	Objective Variable
**CP Input Variation**	*Q_i_*	441W	30% step decrease	Temperature difference of CP
		504 W	20% step decrease	
		567 W	10% step decrease	
		693 W	10% step increase	
		756 W	20% step increase	
		819 W	30% step increase	
**THP Input Variation**	*I*	1A-15A	Increment of 1 A	

**Table 5:** Case arrangement for closed-loop control effect analysis.

Simulation Conditions	Inputs	Description	Control Variable	Objective Variable
Step disturbance	*Q_i_*	10% step increase at 50s	Electric current of THP	Temperature difference of CP
External disturbance		External disturbance between 600-700W within 900–1000 s		
Periodic disturbance		Square wave periodic disturbance with high level of 660 W and low level of 600 W after 200 s		

**Table 6:** Closed-loop control performance parameters for +10% step in Qi.

Controllers	γ(%)	τ(sec)	δ(%)
**Basic PID**	9.4%	68	0.426%
**Fuzzy-PID**	3.81%	16	0.073%
**BP-PID**	4.93%	10	0.033%

**Table 7:** Closed-loop control performance parameters for external disturbance in *Q_i_*.

Controllers	γ(%)	τ(sec)	δ(%)
**Basic PID**	9.48%	86	0.78%
**Fuzzy-PID**	3.81%	16	0.0067%
**BP-PID**	4.93%	10	0.0013%

**Table 8:** Closed-loop control performance parameters for periodic disturbance in *Q_i_*.

Controllers	γ(%)	τ(sec)	δ(%)
**Basic PID**	9.31%	84	4%
**Fuzzy-PID**	3.6%	16	0.573%
**BP-PID**	4.7%	10	0.4%

## Nomenclature

T	Temperature (K).
$T_{m}$	Temperature of cold plate (K).
$T_{fi}$	Temperature of inlet water (K).
$T_{fo}$	Temperature of outlet water (K).
$T_{h}$	Hot-side temperature of THP (K).
$T_{c}$	Cold-side temperature of THP (K).
$m_{cp}$	Mass of cold plate (kg).
$m_{f}$	Mass of water (kg).
$c_{cp}$	Specific heat of cold plate (J∙kg^−1^∙K^−1^).
$c_{f}$	Specific heat of water (J∙kg^−1^∙K^−1^).
$m_{t}c_{t}$	Heat capacity of THP (J/K).
R	Thermal resistance (K/W).
G	Mass flow rate (kg/s).
$Q_{i}$	Thermal load (W).
$Q_{h}$	Heat dissipation (W).
$Q_{c}$	Cooling capacity (W).
P	Power consumption (W).
I	Electric current (A).
$r_{t}$	Electrical resistance (Ω).
$K_{t}$	Thermal conductance of THP (W/K).
$\Delta T_{cp}$	Temperature difference of cold plate (K).
$\Delta T_{cp\_sta}$	Stable value of $\Delta T_{cp}$ (K).
$\Delta T_{cp\_\mathrm{PID}}$	$\Delta T_{cp}$ under the PID control (K).
$\Delta T_{cp\_F}$	$\Delta T_{cp}$ under the fuzzy-PID control (K).
$\Delta T_{cp\_\mathrm{BP}}$	$\Delta T_{cp}$ under the BP-PID control (K).
$\Delta T_{r}$	Desired value of $\Delta T_{cp}$ (K).
$\Delta T_{t}$	Temperature difference of THP (K).
Greek symbols	
$\alpha_{t}$	Seeback coefficient (V/K).
η	Heat exchange efficiency.
*ε*	Coefficient of performance.
τ	Response time (s).
Subscripts	
cp	Cold plate.
f	Working fluids.
t	Thermoelectric cooler.
max	Maximal.
opt	Optimal.

## Appendix A

### Appendix A.2. Fuzzy Control Rules

**Table A1:** (a) Fuzzy control rules of Δ*K_p_*. (b) Fuzzy control rules of Δ*K_i_*. (c) Fuzzy control rules of Δ*K_d_*.

(a)							
***E_i_/CE_j_***	**NB**	**NM**	**NS**	**Z**	**PS**	**PM**	**PB**
NB	PB	PB	PM	PM	PS	Z	Z
NM	PB	PM	PM	PS	PS	Z	NS
NS	PM	PM	PM	PS	Z	NS	NS
Z	PM	PM	PS	Z	NS	NM	NM
PS	PS	PS	Z	NS	NS	NM	NM
PM	PS	Z	NS	NM	NM	NM	NB
PB	Z	Z	NS	NM	NM	NB	NB
(b)							
***E_i_/CE_j_***	**NB**	**NM**	**NS**	**Z**	**PS**	**PM**	**PB**
NB	NB	NB	NM	NM	NS	Z	Z
NM	NB	NB	NM	NS	NS	Z	Z
NS	NB	NM	NS	NS	Z	PS	PS
Z	NM	NM	NS	Z	PS	PM	PM
PS	NM	NS	Z	PS	PS	PM	PM
PM	Z	Z	PS	PS	PS	PB	PB
PB	Z	Z	PS	PM	PM	PB	PB
(c)							
***E_i_/CE_j_***	**NB**	**NM**	**NS**	**Z**	**PS**	**PM**	**PB**
NB	PS	NS	NB	NB	NB	NM	PS
NM	PS	NS	NB	NM	NM	NS	Z
NS	Z	NS	NM	NM	NS	NS	Z
Z	Z	NS	NS	NS	NS	NS	Z
PS	Z	Z	Z	Z	Z	Z	Z
PM	PB	NS	PS	PS	PS	PS	PB
PB	PB	PM	PM	PM	PS	PS	PB

### Appendix A.3. Weight Coefficients Distribution

**Table A2:** Weight coefficients distribution of BP-NN.

Weight Coefficients	Values							
$\omega_{jm}^{\left(2\right)}$	0.1870		0.3630		−0.3492		−0.1723	
	−0.1957		0.4927		−0.3608		−0.1611	
	0.5682		−0.0258		0.0277		0.7030	
	−0.2202		0.0786		0.2271		0.2690	
	0.0357		−0.0086		−0.1074		−0.2447	
$\omega_{mn}^{\left(3\right)}$	0.5776	0.2809		−0.3780		−0.3311		−0.3680
	0.2400	0.2654		−0.1383		0.4525		0.3335
	−0.1706	−0.2657		−0.2839		−0.2201		0.1361

## Appendix B

### Parameter Determination

**Table A3:** Parameters of the simulated controllers.

Controllers	Parameters							
Basic PID controller	*K_p_*	*K_i_*	*K_d_*					
	12	1	0.5					
Fuzzy-PID controller	*K_p_*_0_	*K_i_*_0_	*K_d_*_0_	*k_e_*	*k_ec_*	*k_p_*	*k_i_*	*k_d_*
	12	1	0.5	0.9	0.1	0.3	12	0.1
BP-PID controller	*K_p_*_0_	*K_i_*_0_	*K_d_*_0_	*α*	*β*			
	12	1	0.5	0.7	0.03			

Sample period, *T_s_* = 1.0 s.
